# Epidemiology of Influenza Virus Types and Subtypes in South Africa, 2009–2012^1^

**DOI:** 10.3201/eid2007.131869

**Published:** 2014-07

**Authors:** Adam L. Cohen, Orienka Hellferscee, Marthi Pretorius, Florette Treurnicht, Sibongile Walaza, Shabir Madhi, Michelle Groome, Halima Dawood, Ebrahim Variava, Kathleen Kahn, Nicole Wolter, Anne von Gottberg, Stefano Tempia, Marietjie Venter, Cheryl Cohen

**Affiliations:** Centers for Disease Control and Prevention, Atlanta, Georgia, USA, and Pretoria, South Africa (A.L. Cohen, S. Tempia, M. Venter);; National Institute for Communicable Diseases, Sandringham, South Africa (O. Hellferscee, M. Pretorius, F. Treurnicht, S. Walaza, N. Wolter, A. von Gottberg, S. Tempia, M. Venter, C. Cohen);; University of the Witwatersrand, Johannesburg, South Africa (S. Madhi, M. Groome, N. Wolter, A. von Gottberg, C. Cohen);; Medical Research Council: Respiratory and, Meningeal Pathogens Research Unit, Johannesburg (S. Madhi, M. Groome);; Pietermaritzburg Metropolitan Hospital Complex, Pietermaritzburg, South Africa (H. Dawood);; University of KwaZulu-Natal, Durban, South Africa (H. Dawood);; Klerksdorp Tshepong Hospital, Klerksdorp, South Africa (E. Variava);; University of the Witwatersrand, Johannesburg (K. Kahn);; Umeå University, Umeå, Sweden (K. Kahn);; INDEPTH Network, Accra, Ghana (K. Kahn);; University of Pretoria, Pretoria, (M. Venter)

**Keywords:** influenza, influenza A, influenza B, H3N2, H1N1, types, subtypes, pneumonia, South Africa, viruses

## Abstract

To determine clinical and epidemiologic differences between influenza caused by different virus types and subtypes, we identified patients and tested specimens. Patients were children and adults hospitalized with confirmed influenza and severe acute respiratory illness (SARI) identified through active, prospective, hospital-based surveillance from 2009–2012 in South Africa. Respiratory specimens were tested, typed, and subtyped for influenza virus by PCR. Of 16,005 SARI patients tested, 1,239 (8%) were positive for influenza virus. Patient age and co-infections varied according to virus type and subtype, but disease severity did not. Case-patients with influenza B were more likely than patients with influenza A to be HIV infected. A higher proportion of case-patients infected during the first wave of the 2009 influenza pandemic were 5–24 years of age (19%) than were patients infected during the second wave (9%). Although clinical differences exist, treatment recommendations do not differ according to subtype; prevention through vaccination is recommended.

## Abstract 2

Patient age and co-infections, but not disease severity, were associated with virus type and subtype.

Most influenza in humans is caused by 2 types of influenza virus: A and B. On the basis of the hemagglutinin and neuraminidase proteins on the surface of the virus, influenza A viruses are further subdivided into subtypes, 2 of which have commonly caused disease in humans over the past century: H3N2 and H1N1. The proportion of these 3 types and subtypes of influenza virus—A(H3N2), A(H1N1), and B—that circulate among humans varies each year. In 2009, a novel pandemic strain of influenza A(H1N1) virus, now called influenza A(H1N1)pdm09 virus, became the dominant H1N1 virus strain circulating worldwide (*1*).

It is generally not possible to distinguish infection caused by different influenza types and subtypes by clinical features (*2*,*3*), although differences in severity have been observed (*4*–*6*). Analyses of vital statistics data from the United States and South Africa have suggested that the numbers of excess deaths associated with influenza are higher in years when influenza A(H3N2) virus is circulating than when influenza B or prepandemic influenza A(H1N1) virus is circulating (*4*,*7*). Some studies have suggested that influenza A(H1N1)pdm09 virus infection led to more severe outcomes than did other types and subtypes (*8*,*9*). In the first 3 months after influenza A(H1N1)pdm09 virus was identified in South Africa, 91 deaths among 12,331 patients with laboratory-confirmed cases were identified; rates of HIV infection and pregnancy among those who died were high (*10*). After the influenza pandemic, studies showed that A(H1N1)pdm09 virus was more likely than previously circulating virus types and subtypes to affect children and young adults and that severe disease was associated with clinical characteristics such as obesity (*11*,*12*). The data conflict with regard to whether severity of disease increases with subsequent waves of A(H1N1)pdm09 virus infection (*13**–**17*).

Little data have been reported from Africa on clinical and epidemiologic differences caused by different influenza virus types and subtypes. The objective of our study was 2-fold. First, we sought to compare the demographic and clinical characteristics, factors associated with infection, and disease severity among case-patients hospitalized with severe acute respiratory illness (SARI) associated with influenza A(H1N1)pdm09, A(H3N2), and B viruses in South Africa during 2009–2012. Second, we sought to compare the characteristics of case-patients infected during the first wave of influenza A(H1N1)pdm09 infection in 2009 with those of case-patients infected during the subsequent wave in 2011. Because this surveillance was started in 2009, we did not include prepandemic A(H1N1) virus strains in this study.

## Materials and Methods

### Setting and Time 

The SARI program is an active, prospective, sentinel, hospital-based surveillance system that monitors children and adults hospitalized with pneumonia in 4 provinces in South Africa (*18*). In February 2009, SARI surveillance was implemented in 3 of the 9 provinces of South Africa (Chris Hani-Baragwanath Academic Hospital, an urban site in Gauteng Province; Edendale Hospital, a periurban site in KwaZulu-Natal Province; and Matikwana and Mapulaneng Hospitals, rural sites in Mpumalanga Province). In June 2010, an additional surveillance site was introduced at Klerksdorp and Tshepong Hospitals, periurban sites in Northwest Province. This surveillance, which includes testing for influenza virus and HIV, has received human subjects review and approval by the University of Witswatersrand, South Africa. The US Centers for Disease Control and Prevention deemed this a nonresearch surveillance activity. The study was conducted during 2009–2012.

### Case Definitions and Patient Enrollment

A case of SARI was defined as acute lower respiratory tract infection (or pneumonia) in a patient hospitalized within 7 days of illness onset. Children 2 days through <3 months of age with physician-diagnosed sepsis or acute lower respiratory tract infection (including, for example bronchitis, bronchiolitis, pneumonia, and pleural effusion) and children 3 months through <5 years of age with physician-diagnosed acute lower respiratory tract infection were enrolled. Among patients >5 years of age, we enrolled those who met the World Health Organization case definition of SARI: sudden onset of reported or measured fever (>38°C), cough or sore throat, and shortness of breath or difficulty breathing (*19*).

All patients admitted to a hospital during Monday–Friday were eligible for enrollment in the study; adult patients at Chris Hani-Baragwanath Academic Hospital were systematically sampled 2 of every 5 working days per week. Patients were enrolled within the first 24 hours of admission. We determined the number of patients who were admitted, met study case definitions, and were enrolled. Study staff were centrally trained and completed case report forms until discharge for all enrolled patients; staff collected respiratory (nasopharyngeal) aspirates from patients <5 years of age and nasopharyngeal and throat swab specimens from patients >5 years of age and blood specimens from consenting patients. Patients were admitted to an intensive care unit, and specimens for bacterial culture and tuberculosis testing were collected at the discretion of the attending physician. For children <5 years of age, we gathered data on additional clinical signs and symptoms; for adolescents and adults >12 years of age, we gathered information on smoking and alcohol use. Informed consent was obtained for all enrollment, laboratory testing, and anonymized, linked HIV testing.

### Laboratory Methods

Respiratory specimens were placed in viral transport media, kept at 4–8°C, and sent to the National Institute for Communicable Diseases in Johannesburg within 72 hours of collection. Respiratory specimens were tested by multiplex real-time reverse transcription PCR for 10 respiratory viruses (influenza A and B viruses; parainfluenza viruses 1, 2, and 3; respiratory syncytial virus; enterovirus; human metapneumovirus; adenovirus; and rhinovirus) (*20*). Influenza-positive specimens were subtyped by using the Centers for Disease Control and Prevention real-time reverse transcription PCR protocol for detection and characterization of influenza virus (*21*). *Streptococcus pneumoniae* was identified by quantitative real-time PCR that detected the *lytA* gene from whole-blood specimens (*22*). When available, data on HIV infection status were obtained through routine standard-of-care testing at the treating hospital. When those data were not available, HIV testing was implemented at the National Institute for Communicable Diseases through anonymized, linked, dried blood-spot specimen testing by HIV PCR for children <18 months of age and by ELISA for patients >18 months of age.

### Statistical Analyses

We excluded from the analysis influenza virus–positive case-patients for whom subtyping could not be performed because of low concentration of virus. Univariate comparisons were performed by using multinomial or logistic regression. We conducted multinomial regression to compare demographic and clinical characteristics, associated factors, and disease severity among patients infected with the 3 influenza types and subtypes. Multinomial regression enables modeling of outcome variables with >2 categories and relates the probability of being in a category (in this instance either influenza A[H3N2] or B virus) to the probability of being in a baseline category (in this instance influenza [H3N2] virus). A complete set of coefficients are estimated for each of the categories being compared with the baseline, and the effect of each predictor in the model is measured as relative risk ratio (RRR). For this analysis, we used the influenza virus A(H3N2)–infected group as the baseline category because influenza A(H3N2) virus is considered to induce more severe illness (*4*,*7*). We conducted 2 logistic regression models to compare patients infected with influenza A with those infected with influenza B and to compare patients infected during the first wave of influenza A(H1N1)pdm09 with patients infected during subsequent waves of influenza A(H1N1)pdm09. All models were built by using stepwise forward selection. Covariates for which p value was <0.2 at the univariate analysis were assessed for significance with multivariable analysis, and statistical significance was assessed at p<0.05 for all multivariable models. We assessed 2-way interactions by inclusion of product terms for all variables remaining in the final models. Additional modeling is shown in the Technical Appendix.

## Results

From February 2009 through December 2012, a total of 21,792 patients hospitalized with lower respiratory tract infection were approached for enrollment in SARI surveillance. Of those, 16,005 (73%) were enrolled and 1,239 (8%) had positive influenza virus test results. Of the 5,876 patients who were approached but not enrolled, the most common reasons for not enrolling were unavailability of a legal guardian (among children <5 years of age; 1,452 [25%]), refusal (1,296 [22%]), and being confused or too ill (431 [7%]). Of the influenza-positive SARI cases, 463 (37%) were caused by influenza A(H3N2), 338 (27%) by influenza A(H1N1)pdm09, and 418 (34%) by influenza B viruses; 20 (2%) influenza A viruses could not be further subtyped because of low viral yield in the samples. Influenza epidemics occur annually during the colder months in South Africa (May–September), and little activity occurs during the rest of the year (Figure). The circulating types and subtypes varied between study years and within annual epidemics. During 2009, influenza virus activity occurred in 2 peaks; the first was caused by subtype A(H3N2) (194/379, 51%), which occurred earlier than in the other years, and the second was caused by subtype A(H1N1)pdm09 (160/379 42%) (Table 1 [an expanded version of this table is shown in the Technical Appendix]; Figure). The predominant influenza virus types or subtypes in the other years were as follows: B (164/273, 60%) in 2010, A(H1N1)pdm09 (140/362, 39%) in 2011, and A(H3N2) (99/205, 48%) and B (105/205, 51%) in 2012. Most (71%) case-patients were at Chris Hani-Baragwanath Academic Hospital, which reflects the higher number of SARI case-patients enrolled there. Of 12,494 SARI case-patients for whom treatment data were available, 7 (0.1%) received oseltamivir, 1 of whom had laboratory-confirmed influenza. Of 12,173 SARI case-patients for whom influenza vaccine histories were available, 19 (0.2%) reported having been vaccinated. HIV test results were available for 947 (76%) of influenza case-patients. Of those, 399 (42%) were positive for HIV: 377 (94%) from anonymized testing at the National Institute for Communicable Diseases and 22 (6%) from standard-of-care testing at the treating hospitals.

**Figure:**
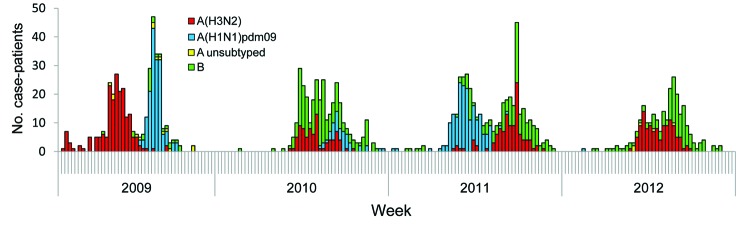
Number of case-patients hospitalized with influenza-associated severe acute respiratory illness, by week and virus strain at 4 sites, South Africa, 2009–2012.

**Table 1:** Characteristics of patients hospitalized with influenza-associated severe acute respiratory illness, by virus type and subtype, 4 sites, South Africa, 2009–2012*

Characteristic	Influenza type and subtype

A(H3N2) (reference)		A(H1N1)pdm09			B	
No. pos/no. tested (% pos)		No. pos/no. tested (% pos)	Adjusted RRR (95% CI)		No. pos/no. tested (% pos)	Adjusted RRR (95% CI)
Age group, y							
<5	265/463 (57.2)		167/338 (49.4)	Reference		171/418 (40.9)	Reference
5–24	35/463 (7.6)		49/338 (14.5)	2.3 (1.4–3.8)		43/418 (10.3)	2.0 (1.2–3.4)
25–44	96/463 (20.7)		78/338 (23.1)	1.3 (0.9–2.0)		128/418 (30.6)	1.5 (1.0–2.1)†
45–64	44/463 (9.5)		35/338 (10.4)	1.4 (0.9–2.4)		55/418 (13.2)	1.4 (0.9–2.2)
>65	23/463 (5.0)		9/338 (2.7)	0.6 (0.2–1.3)		21/418 (5.0)	1.1 (0.6–2.2)
Male	207/461 (44.9)		149/336 (44.4)			177/417 (42.5)	
Black African	452/460 (98.3)		327/336 (97.3)			407/416 (97.8)	
Year							
2009	194/463 (41.9)		160/338 (47.3)	Reference		25/418 (6.0)	Reference
2010	72/463 (15.6)		37/338 (11.0)	0.6 (0.4–1.0)†		164/418 (39.2)	16.8 (10.1–27.9)
2011	98/463 (21.2)		140/338 (41.4)	1.7 (1.2–2.5)		124/418 (29.7)	9.5 (5.7–15.6)
2012	99/463 (21.4)		1/338 (0.3)	0.0 (0.0–0.1)		105/418 (25.1)	7.8 (4.7–13.0)
Co-infections and underlying medical conditions							
HIV infection	112/311 (36.0)		110/271 (40.6)			170/352 (48.3)	
Tuberculosis	42/458 (9.2)		34/335 (10.2)			38/411 (9.3)	
Underlying medical condition excluding tuberculosis, HIV‡	34/460 (7.4)		31/336 (9.2)			38/417 (9.1)	
Pregnancy	3/251 (1.2)		2/187 (1.1)			3/24 (1.3)	
Pneumococcal co-infection detected by PCR	23/310 (7.4)		25/286 (8.7)			32/325 (9.9)	
Clinical presentation and course							
Temperature >38°C	181/364 (49.7)		141/287 (49.1)			138/407 (33.9)	
Cough§	255/264 (96.6)		162/167 (97.0)			163/170 (95.9)	
Tachypnea§	99/250 (39.6)		73/161 (45.3)			62/159 (39.0)	
Difficulty breathing§	188/264 (71.2)		125/167 (74.9)			111/170 (65.3)	
Chest wall indrawing§	96/264 (36.4)		77/167 (46.1)			56/170 (32.9)	
Stridor§	30/264 (11.4)		20/167 (12.0)			36/170 (21.2)	
Symptoms ≥3 d before admission	206/452 (45.6)		153/335 (45.7)			239/415 (57.6)	
Admitted to ICU	4/457 (0.9)		3/336 (0.9)			4/411 (1.0)	
Mechanical ventilation needed	3/457 (0.7)		1/336 (0.3)			4/411 (1.0)	
Supplemental oxygen needed	138/457 (30.2)		117/336 (34.8)			144/411 (35.0)	
Antimicrobial drugs prescribed on admission	402/421 (95.7)		321/335 (95.8)			384/395 (97.2)	
Hospitalized for >2 d	319/451 (70.7)		255/332 (76.8)			323/407 (79.4)	
No. deaths/no. patients (case-fatality ratio)	13/459 (2.8)		5/334 (1.5)			16/412 (3.9)	

*Pos, positive; RRR, relative risk ratio; ICU, intensive care unit. An expanded version of this table is available in the Technical Appendix. †p<0.05. ‡Asthma, other chronic lung disease, chronic heart disease (valvular heart disease, coronary artery disease, or heart failure excluding hypertension), liver disease (cirrhosis or liver failure), renal disease (nephrotic syndrome, chronic renal failure), diabetes mellitus, immunocompromising conditions excluding HIV infection (organ transplant, immunosuppressive therapy, immunoglobulin deficiency, malignancy), neurologic disease (cerebrovascular accident, spinal cord injury, seizures, neuromuscular conditions), or pregnancy. Concurrent conditions were considered absent for patients for whom the medical records stated that the patient had no underlying medical condition or when there was no direct reference to that condition. §Patients <5 y of age.

The age distribution of SARI case-patients with influenza was bimodal: most of the 1,239 influenza case-patients were <5 years of age (613 [49.5%]), followed by those 25–44 years of age (306 [24.7%]); few patients were ≥65 years of age (53 [4.3%]). This bimodal age distribution is repeated for each of the types and subtypes (Table 1) except that the first wave of A(H1N1)pdm09 infection disproportionately affected those 5–24 years of age (Table 2). According to univariate analysis, case-patients infected with influenza A(H1N1)pdm09 virus were less likely than case-patients infected with influenza A(H3N2) virus to be co-infected with another virus (crude RRR [cRRR] 0.6, 95% CI 0.4–0.8), and case-patients infected with influenza B virus were more likely to be infected with HIV (cRRR 1.7, 95% CI 1.2–2.3), have stridor (cRRR 2.1, 95% CI 1.2–3.6), have symptoms >3 days before admission (cRRR 1.6, 955 CI 1.2–2.1), and to have been hospitalized for >2 days (cRRR 1.6, 95% CI 1.2–2.2), and were less likely to have a measured fever of >38°C (cRRR 0.5, 95% CI 0.4–0.7) (Table 1). In the multivariate analysis model, only age and year remained statistically significant (Table 1). We found no statistical difference in case-fatality rates between virus types and subtypes (2.8% for A[H3N2], 1.5% for A[H1N1]pdm09, and 3.9% for B) and no difference in other markers of severity, such as admission to an intensive care unit, need for mechanical ventilation, need for supplemental oxygen, or prolonged hospitalization (Table 1).

**Table 2:** Characteristics of patients hospitalized with influenza A(H1N1)pdm09-associated severe acute respiratory illness, by wave, 4 sites, South Africa, 2009–2012*

Characteristic	A(H1N1)pdm09	Crude OR (95% CI)	Adjusted OR (95% CI)

First wave (2009), no. pos/no. tested (% pos)	Second wave (2011), no. pos/no. tested (% pos)
Age group, y				
<5	87/160 (54.4)	67/140 (47.9)	Reference	Reference
5–24	31/160 (19.4)	12/140 (8.6)	0.5 (0.2–1.1)	0.6 (0.3–1.4)
25–44	24/160 (15.0)	42/140 (30.0)	2.3 (1.3–4.1)	2.8 (1.5–5.1)
45–64	13/160 (8.1)	16/140 (11.1)	1.6 (0.7–3.6)	2.0 (0.9–4.6)
>65	5/160 (3.1)	3/139 (2.1)	0.8 (0.2–3.4)	1.1 (0.3–5.1)
Male	76/159 (47.8)	57/139 (41.1)	0.8 (0.5–1.2)	
Black African	156/159 (98.1)	135/139 (97.1)	1.5 (0.3–7.0)	
Site				
Soweto	104/160 (65.0)	98/140 (70.0)	Reference	Reference
Klerksdorp	Not applicable	12/140 (8.6)	Not calculated	Not calculated
Pietermaritzburg	6/160 (3.8)	12/140 (8.6)	0.4 (0.2–0.7)	0.4 (0.2–0.8)
Agincourt	50/160 (31.3)	18/140 (12.9)	2.1 (0.8–5.9)	2.7 (0.96–7.8)
Co-infections and underlying medical conditions				
HIV-infected	47/119 (39.5)	49/117 (41.9)	1.1 (0.7–1.9)	
Tuberculosis	18/158 (11.4)	12/139 (8.6)	0.7 (0.3–1.6)	
Underlying medical condition excluding tuberculosis, HIV	11/159 (6.9)	15/139 (10.8)	1.6 (0.7–3.7)	
Pregnancy	2/83 (2.4)	0/82 (0)	Not calculated	
Bacterial/viral respiratory co-infection				
Pneumococcal co-infection detected by PCR	15/129 (11.6)	7/127 (5.5)	0.4 (0.2–1.1)	
Respiratory syncytial virus	2/153 (1.3)	11/140 (7.9)	6.4 (1.4–29.6)	
Adenovirus	0/153 (0)	18/140 (12.9)	Not calculated	
Parainfluenzavirus 1, 2, or 3	10/160 (6.3)	3/140 (2.1)	0.3 (0.1–1.2)	
Human metapneumovirus	6/153 (3.9)	1/140 (0.7)	0.2 (0.0–1.5)	
Rhinovirus	16/153 (10.5)	11/140 (7.9)	0.7 (0.3–1.6)	
Enterovirus	2/153 (1.3)	2/140 (1.4)	1.1 (0.2–7.9)	
Clinical presentation and course				
Temperature ≥38°C	76/110 (69.1)	46/139 (33.1)	0.2 (0.1–0.4)	
Cough†	83/87 (95.4)	66/67 (98.5)	3.2 (0.3–29.1)	
Tachypnea†	32/84 (38.1)	34/65 (52.3)	1.8 (0.9–3.4)	
Difficulty breathing†	69/87 (79.3)	45/67 (67.2)	0.5 (0.3–1.1)	
Chest wall indrawing†	44/87 (50.6)	25/67 (47.3)	0.6 (0.3–1.1)	
Stridor†	4/87 (4.6)	11/67 (16.4)	4.1 (1.2–13.4)	
Tachycardia†	44/87 (50.6)	43/67 (64.2)	1.8 (0.9–3.4)	
Diarrhea†	16/87 (18.4)	9/67 (13.4)	0.7 (0.3–1.7)	
Unable to eat†	29/87 (33.3)	11/67 (6.4)	0.4 (0.2–0.9)	
Vomiting †	26/87 (29.9)	22/67 (32.8)	1.1 (0.6–2.3)	
Lethargy†	19/87 (21.8)	10/67 (14.9)	0.6 (0.3–1.5)	
Symptoms ≥3 d before admission	58/158 (36.7)	74/139 (53.2)	2.0 (1.2–3.1)	
Admission to intensive care unit	1/159 (1.0)	2/139 (1.4)	2.3 (0.2–25.7)	
Mechanical ventilation needed	0/159 (0)	1/139 (1.0)	Not calculated	
Supplemental oxygen needed	37/159 (23.3)	61/139 (43.9)	2.6 (1.6–4.2)	
Antimicrobial drugs prescribed on admission	151/158 (95.6)	134/139 (96.4)	1.2 (0.4–4.0)	
Duration of hospitalization >2 d	117/157 (74.5)	107/138 (77.5)	1.2 (0.7–2.0)	
No. deaths/no. patients (case-fatality ratio)	2/158 (1.3)	2/138 (1.5)	1.1 (0.2–8.3)	

*Pos, positive; OR, odds ratio. †Patients <5 y of age.

To further explore the association between influenza types and characteristics such as HIV status, we conducted a univariate analysis and constructed a multivariable logistic regression model comparing influenza B virus with influenza A (both A[H3N2] and A[H1N1]pdm09) viruses. Except for co-infection with any virus other than influenza, the same variables were significant on this univariate analysis as were significant on the previous analysis. According to multivariate analysis, only year and HIV status remained statistically significant and were retained in the final model. Because age group was not significantly associated with virus type and did not have an interaction with HIV infection in the multivariate model, we did not include age in the final model. When we controlled for year, this model showed that case-patients with influenza B virus infection were more likely than patients with influenza A virus infection to also be infected with HIV (adjusted odds ratio 1.4, 95% CI 1.02–1.80).

According to univariate analysis, case-patients in the second wave of the A(H1N1)pdm09 pandemic were less likely than case-patients in the first wave to have had a measured fever of >38°C (crude odds ratio [cOR] 0.2, 95% CI 0.1–0.4) and more likely to have been co-infected with respiratory syncytial virus (cOR 6.4, 95% CI 1.4–29.6), have had symptoms for >3 days at admission (cOR 2.0, 95% CI 1.2–3.1), and to have needed supplemental oxygen (cOR 2.6, 95% CI 1.6–4.2; Table 2). According to multivariable logistic regression, only age group and surveillance site remained statistically significant (Table 2). Severity of hospitalization, as measured by admission to an intensive care unit, need for mechanical ventilation, need for supplemental oxygen, or prolonged hospitalization, did not differ between waves (Table 2). In addition, case-fatality rates did not differ between the first (1.3%) and second (1.5%) waves.

## Discussion

The influenza virus types and subtypes that circulated during the annual winter influenza seasons in South Africa varied from 2009 (the year of the A(H1N1)pdm09 pandemic) to 2012. Characteristics of patients hospitalized with SARI differed by infection with different influenza types and subtypes, particularly with regard to age and co-infection with HIV. In South Africa, the age distribution of those hospitalized with influenza during the second wave of the A(H1N1)pdm09 pandemic was more similar to the age distribution of those infected by seasonal influenza types and subtypes (a bimodal distribution with a peak in young adults 25–44 years of age) than to that of those who experienced severe disease during the first wave of the A(H1N1)pdm09 pandemic (*18*). This age distribution of respiratory influenza infection in South Africa is driven by the high prevalence of HIV infection among young adults in South Africa because HIV-infected adults are at increased risk for severe disease from influenza virus infection (*18*). In South Africa in 2009, the prevalence of HIV infection among the total population was 11% (*23*) and the prevalence among women attending antenatal care was 29% (*24*). In other settings, infection with influenza B virus is associated with less severe disease than is infection with influenza A(H3N2) virus (*4*–*6*). We found that hospitalization with influenza B virus infection was associated with HIV infection. This finding suggests that underlying immunosuppression can trigger severe influenza illness requiring hospitalization for infection caused by virus types, such as influenza B, that can cause milder illness in immunocompetent persons.

Unlike case-fatality rates and disease severity previously reported from South Africa and other countries, we found no differences in case-fatality rates or severity in South Africa during the years studied among the virus types and subtypes or between the first and second waves of the A(H1N1)pdm09 pandemic. Previous excess death models have suggested increased deaths in years when influenza A(H3N2) virus circulated in South Africa (*7*). The contrast between case-fatality and severity found in this analysis and that observed in previous studies in South Africa might be the result of different methods or different study periods. Although our study was conducted over fewer years and might have had less power to detect differences at a population level, we were able to look at markers of severity in individual cases and to compare different waves of A(H1N1)pdm09 virus infection.

This study has several limitations. We compared influenza types and subtypes across 4 years, so some associations might have resulted from changes in prevalence of other diseases such as HIV over the same period. We do not have data on nonrespiratory influenza disease, which might have different associations with influenza virus types and subtypes than respiratory influenza disease. Although obesity and pregnancy have been associated with infection with influenza A(H1N1)pdm09 virus, we identified few case-patients who were pregnant, and obesity was not included in our analysis because so few obese case-patients were identified by surveillance. Other factors and conditions, such as neuromuscular disorders that are associated with severe influenza disease, might be associated with specific types and subtypes, but we were unable to evaluate this association because of the small number of patients with these conditions. Patients were not enrolled on weekends, which could introduce bias if patients had more or less severe disease on weekends than patients enrolled during the week. Last, most patients were identified at a single surveillance site, so the results might more strongly reflect differences observed at that site.

Vaccination remains the best way to prevent influenza infection. Influenza vaccination coverage is very low in South Africa (*25*). In that country, influenza vaccination is recommended for HIV-infected persons (*26*), and efforts should be made to encourage higher vaccine coverage. Although differences exist between infection with different influenza types and subtypes, particularly with regard to age distribution and co-infections, it can be difficult for the clinician to differentiate infection by different types and subtypes for individual patients. Current treatment recommendations do not differ according to the subtype with which a patient is infected, in part because it is not common to type and subtype the virus in individual patients in time for clinical decision-making. 

Technical Appendix Additional modeling of influenza A and B virus, HIV status, and patient age and characteristics of patients hospitalized with influenza-associated severe acute respiratory illness, by virus type and subtype, 4 sites, South Africa, 2009–2012.

## References

[1] Novel Swine-Origin Influenza A. (H1N1) Virus Investigation Team; Dawood FS, Jain S, Finelli L, Shaw MW, Lindstrom S, Garten RJ, et al. Emergence of a novel swine-origin influenza A (H1N1) virus in humans. N Engl J Med. 2009;360:2605–15. 10.1056/NEJMoa0903810 19423869

[2] Paul Glezen W, Schmier J, Kuehn C, Ryan K, Oxford J. The burden of influenza B: a structured literature review. Am J Public Health. 2013;103:e43–51. 10.2105/AJPH.2012.301137 23327249 PMC3673513

[3] Irving SA, Patel DC, Kieke BA, Donahue JG, Vandermause MF, Shay DK, Comparison of clinical features and outcomes of medically attended influenza A and influenza B in a defined population over four seasons: 2004–2005 through 2007–2008. Influenza Other Respir Viruses. 2012;6:37–43. 10.1111/j.1750-2659.2011.00263.x PMC4941556 21668663

[4] Thompson WW, Shay DK, Weintraub E, Brammer L, Cox N, Anderson LJ, Mortality associated with influenza and respiratory syncytial virus in the United States. JAMA. 2003;289:179–86. 10.1001/jama.289.2.179 12517228

[5] Esposito S, Molteni CG, Daleno C, Valzano A, Fossali E, Da Dalt L, Clinical and socioeconomic impact of different types and subtypes of seasonal influenza viruses in children during influenza seasons 2007/2008 and 2008/2009. BMC Infect Dis. 2011;11:271. 10.1186/1471-2334-11-271 21992699 PMC3205059

[6] McCullers JA, Hayden FG. Fatal influenza B infections: time to reexamine influenza research priorities. J Infect Dis. 2012;205:870–2. 10.1093/infdis/jir865 22291194

[7] Cohen C, Simonsen L, Kang JW, Miller M, McAnerney J, Blumberg L, Elevated influenza-related excess mortality in South African elderly individuals, 1998–2005. Clin Infect Dis. 2010;51:1362–9. 10.1086/657314 21070141 PMC3106243

[8] Chaves SS, Aragon D, Bennett N, Cooper T, D'Mello T, Farley M, Patients hospitalized with laboratory-confirmed influenza during the 2010–2011 influenza season: exploring disease severity by virus type and subtype. J Infect Dis. 2013;208:1305–14. 10.1093/infdis/jit316 23863950

[9] Kawai N, Ikematsu H, Kawashima T, Maeda T, Ukai H, Hirotsu N, Increased symptom severity but unchanged neuraminidase inhibitor effectiveness for A(H1N1)pdm09 in the 2010–2011 season: comparison with the previous season and with seasonal A(H3N2) and B. Influenza Other Respir Viruses. 2013;7:448–55. 10.1111/j.1750-2659.2012.00421.x PMC5779821 22897904

[10] Archer B, Cohen C, Naidoo D, Thomas J, Makunga C, Blumberg L, Interim report on pandemic H1N1 influenza virus infections in South Africa, April to October 2009: epidemiology and factors associated with fatal cases. Euro Surveill. 2009;14:19369. 19883549 10.2807/ese.14.42.19369-en

[11] To KK, Wong SS, Li IW, Hung IF, Tse H, Woo PC, Concurrent comparison of epidemiology, clinical presentation and outcome between adult patients suffering from the pandemic influenza A (H1N1) 2009 virus and the seasonal influenza A virus infection. Postgrad Med J. 2010;86:515–21. 10.1136/pgmj.2009.096206 20693151 PMC10017011

[12] Morgan OW, Bramley A, Fowlkes A, Freedman DS, Taylor TH, Gargiullo P, Morbid obesity as a risk factor for hospitalization and death due to 2009 pandemic influenza A(H1N1) disease. PLoS ONE. 2010;5:e9694. 10.1371/journal.pone.0009694 20300571 PMC2837749

[13] Lehners N, Geis S, Eisenbach C, Neben K, Schnitzler P. Changes in severity of influenza A(H1N1) pdm09 infection from pandemic to first postpandemic season, Germany. Emerg Infect Dis. 2013;19:748–55. 10.3201/eid1905.130034 23697801 PMC3647517

[14] Ramakrishna K, Peter JV, Karthik G, Abraham AM, Surekha V, Karthik R, Influenza A (H1N1) 2009 pandemic: was there a difference in the two waves in patients requiring admission to the intensive-care unit? Clin Microbiol Infect. 2011;17:1355–8. 21679373 10.1111/j.1469-0691.2011.03584.x

[15] Altmann M, Fiebig L, Buda S, von Kries R, Dehnert M, Haas W. Unchanged severity of influenza A(H1N1)pdm09 infection in children during first postpandemic season. Emerg Infect Dis. 2012;18:1755–62. 10.3201/eid1811.120719 23092713 PMC3559159

[16] Truelove SA, Chitnis AS, Heffernan RT, Karon AE, Haupt TE, Davis JP. Comparison of patients hospitalized with pandemic 2009 influenza A (H1N1) virus infection during the first two pandemic waves in Wisconsin. J Infect Dis. 2011;203:828–37. 10.1093/infdis/jiq117 21278213 PMC3071126

[17] Delgado-Rodriguez M, Castilla J, Godoy P, Martin V, Soldevila N, Alonso J, Different prognosis in hospitalized patients with influenza one season after the pandemic H1N1 influenza of 2009–2010 in Spain. Influenza Other Respi Viruses. 2013;7:1336–42. 10.1111/irv.12119 23647645 PMC4634253

[18] Cohen C, Moyes J, Tempia S, Groom M, Walaza S, Pretorius M, Severe influenza-associated lower respiratory tract infection in a high HIV-prevalence setting, South Africa, 2009–2011. Emerg Infect Dis. 2013;19:1766–74 . 24209781 10.3201/eid1911.130546 PMC3837669

[19] World Health Organization. WHO interim global epidemiological surveillance standards for influenza. July 2012 [cited 2013 Feb 14]. http://who.int/influenza/resources/documents/influenza_surveillance_manual/en/

[20] Pretorius MA, Madhi SA, Cohen C, Naidoo D, Groome M, Moyes J, Respiratory viral coinfections identified by a 10-plex real-time reverse-transcription polymerase chain reaction assay in patients hospitalized with severe acute respiratory illness–South Africa, 2009–2010. J Infect Dis. 2012;206(Suppl 1):S159–65. 10.1093/infdis/jis538 23169964

[21] Centers for Disease Control and Prevention. CDC realtime RTPCR (rRTPCR) protocol for detection and characterization of swine influenza (version 2009). Ref. no. I-007–05. Atlanta: The Centers; 2009.

[22] Carvalho MG, Tondella ML, McCaustland K, Weidlich L, McGee L, Mayer LW, Evaluation and improvement of real-time PCR assays targeting *lytA, ply*, and *psaA* genes for detection of pneumococcal DNA. J Clin Microbiol. 2007;45:2460–6. 10.1128/JCM.02498-06 17537936 PMC1951257

[23] Actuarial Society of South Africa. ASSA 2008 model [cited 2013 Feb 21]. http://aids.actuarialsociety.org.za/ASSA2008-Model-3480.htm

[24] National Department of Health. National Antenatal Sentinel HIV and Syphilis Prevalence Survey in South Africa, 2009. Pretoria (South Africa): The Department; 2009.

[25] Ramkrishna W, Hlungwani P, Furumele T, Nemungadi T, Gumede S, Shilakwe D, Coverage of high-risk groups for influenza vaccination in South Africa, 2011–2013 influenza seasons. Poster presented at: Options for the Control of Influenza; 2013 Sep 5–9; Cape Town, South Africa.

[26] Schoub BD. Recommendations pertaining to the use of viral vaccines: influenza 2012. S Afr Med J. 2012;102:73. 22310432 10.7196/samj.5401

