# Supplementary material for: Epidemiology of Influenza Virus Types and Subtypes in South Africa, 2009–2012
Source: Emerg Infect Dis. 2014 Jul;20(7):1162–9. doi: 10.3201/eid2007.131869 (PMC4073865; doi:10.3201/eid2007.131869)
Supplement: Technical Appendix — Additional modeling of influenza A and B virus, HIV status, and patient age and characteristics of patients hospitalized with influenza-associated severe acute respiratory illness, by virus type and subtype, 4 sites, South Africa, 2009–2012. [file 13-1869-Techapp-s1.pdf]

Article DOI: http://dx.doi.org/10.3201/eid2007.131869

# Epidemiology of Influenza Virus Types and Subtypes in South Africa, 2009–2012

## Technical Appendix

### Additional Modeling of Influenza A and B Virus, HIV Status, and Patient Age

We found an association between age and influenza type and subtype in the multinomial model comparing influenza A(H3N2), A(H1N1)pdm09, and B. The association was significant in the comparison between A(H3N2) and A(H1N1)pdm09 but not when comparing A(H3N2) and B. This is probably due to the age shift of cases observed in South Africa and elsewhere during the first wave of A(H1N1)pdm09 circulation in 2009. In the logistic regression model to assess factors associated with influenza A (both A(H3N2) and A(H1N1)pdm09) and B, age did not have the same association. This is probably due to the fact that the age distribution of cases during the second wave (2011) of A(H1N1)pdm09 in South Africa was similar to those of A(H3N2) and B. When combining A(H3N2) and A(H1N1)pdm09 over the study period into the same category (all influenza A), the effect of the age shift during the first wave of A(H1N1)pdm09 circulation may be missed. On multivariate analysis, only year and HIV status remained statistically associated with influenza types and were retained in the final model (model presented in the text, which we will refer to as model 1). Age group was not significantly associated with influenza types nor had an interaction with HIV infection in the multivariate model, so we did not include age in the final model. Our final model (model 1) showed that, controlling for year, HIV infection was associated with influenza B compared to influenza A infection (adjusted odds ratio [aOR] 1.4, 95% CI 1.02–1.8). However, to explore this association further, since age may still have a confounding effect, we constructed additional models. In a model including year and age (model 2), age was not statistically significant (compared with <5 year olds, 5–24 year olds aOR 1.4, 95% CI 0.9–2.3; 25–44 year olds aOR 1.3, 95% CI 0.95–1.8; 45–64 year olds aOR 1.2, 0.8–1.8; and ≥65 year olds aOR 1.4, 0.8–2.7). In a model with year, age, and HIV infection (model 3), neither age (compared with <5 year olds, 5–24 year olds aOR 1.5, 95% CI 0.9–2.7; 25–44 year olds aOR 1.2, 95% CI 0.8–1.9; 45–64 year olds aOR 1.1, 0.7–

1.8; and ≥65 year olds aOR 1.5, 0.8–2.9) nor HIV infection (aOR 1.3, 95% CI 0.9–1.9) are statistically significant. However, the magnitude and direction of the adjusted odds ratio for HIV infection in model 3 are similar to those in model 1. We interpret this to mean that age is not correlated with influenza A versus B, and we are likely not powered to maintain the statistically significant association between HIV infection and influenza types when we also control for age. Of note, another study in South Africa using the same surveillance system found an association with influenza type and HIV when adjusting for age (*1*).

**Reference**

1. Cohen C, Moyes J, Tempia S, Groom M, Walaza S, Pretorius M, et al. Severe influenza-associated lower respiratory tract infection in a high HIV-prevalence setting-South Africa, 2009–2011. Emerg Infect Dis. 2013;19:1766–74. PubMed

Technical Appendix Table. Characteristics of patients hospitalized with influenza-associated severe acute respiratory illness, by virus type and subtype—4 sites, South Africa, 2009–2012*

| Characteristic | Influenza type and subtype | | | | | | |
|---|---|---|---|---|---|---|---|
| | A(H3N2) (reference) | A(H1N1)pdm09 | | | B | | |
| | No. pos/no. tested (% pos) | No. pos/no. tested (% pos) | Crude RRR (95% CI) | Adjusted RRR (95% CI) | No. pos/no. tested (% pos) | Crude RRR (95% CI) | Adjusted RRR (95% CI) |
| Age group, y | | | | | | | |
| <5 | 265/463 (57.2) | 167/338 (49.4) | Reference | Reference | 171/418 (40.9) | Reference | Reference |
| 5–24 | 35/463 (7.6) | 49/338 (14.5) | 2.2 (1.4–3.6) | 2.3 (1.4–3.8) | 43/418 (10.3) | 1.9 (1.2–3.1) | 2.0 (1.2–3.4) |
| 25–44 | 96/463 (20.7) | 78/338 (23.1) | 1.3 (0.9–1.8) | 1.3 (0.9–2.0) | 128/418 (30.6) | 2.1 (1.5–2.9) | 1.5 (1.0–2.1)† |
| 45–64 | 44/463 (9.5) | 35/338 (10.4) | 1.3 (0.8–2.0) | 1.4 (0.9–2.4) | 55/418 (13.2) | 1.9 (1.2–3.0) | 1.4 (0.9–2.2) |
| ≥65 | 23/463 (5.0) | 9/338 (2.7) | 0.6 (0.3–1.4) | 0.6 (0.2–1.3) | 21/418 (5.0) | 1.4 (0.8–2.6) | 1.1 (0.6–2.2) |
| Male | 207/461 (44.9) | 149/336 (44.4) | 1.0 (0.7–1.3) | | 177/417 (42.5) | 0.9 (0.7–1.2) | |
| Black African | 452/460 (98.3) | 327/336 (97.3) | 1.6 (0.6–4.1) | | 407/416 (97.8) | 1.2 (0.5–3.3) | |
| Site | | | | | | | |
| Soweto | 337/463 (72.8) | 235/338 (69.5) | Reference | | 292/418 (69.9) | Reference | |
| Agincourt | 77/463 (16.6) | 70/338 (20.7) | 1.3 (0.9–1.9) | | 55/418 (13.2) | 0.8 (0.6–1.2) | |
| Pietermaritzburg | 28/463 (6.1) | 20/338 (5.9) | 1.0 (0.6–1.9) | | 43/418 (10.3) | 1.8 (1.1–2.9) | |
| Klerksdorp | 21/463 (4.5) | 13/338 (3.9) | 0.9 (0.6–0.8) | | 28/418 (6.7) | 1.5 (0.9–2.8) | |
| Year | | | | | | | |
| 2009 | 194/463 (41.9) | 160/338 (47.3) | Reference | Reference | 25/418 (6.0) | Reference | Reference |
| 2010 | 72/463 (15.6) | 37/338 (11.0) | 0.6 (0.4–1.0)† | 0.6 (0.4–1.0)† | 164/418 (39.2) | 17.7 (10.7–29.1) | 16.8 (10.1–27.9) |
| 2011 | 98/463 (21.2) | 140/338 (41.4) | 1.7 (1.2–2.4) | 1.7 (1.2–2.5) | 124/418 (29.7) | 9.8 (6.0–16.1) | 9.5 (5.7–15.6) |
| 2012 | 99/463 (21.4) | 1/338 (0.3) | 0 (0–0.1) | 0.0 (0.0–0.1) | 105/418 (25.1) | 8.2 (5.0–13.6) | 7.8 (4.7–13.0) |
| Co-infections and underlying medical conditions | | | | | | | |
| HIV infection | 112/311 (36.0) | 110/271 (40.6) | 1.2 (0.9–1.7) | | 170/352 (48.3) | 1.7 (1.2–2.3) | |
| Tuberculosis | 42/458 (9.2) | 34/335 (10.2) | 1.1 (0.7–1.8) | | 38/411 (9.3) | 1.0 (0.6–1.6) | |
| Underlying medical conditions excluding tuberculosis, HIV‡ | 34/460 (7.4) | 31/336 (9.2) | 1.3 (0.8–2.1) | | 38/417 (9.1) | 1.3 (0.8–2.0) | |
| Pregnancy | 3/251 (1.2) | 2/187 (1.1) | 0.9 (0.1–5.4) | | 3/24 (1.3) | 1.0 (0.2–5.2) | |
| Pneumococcal co-infection detected by PCR | 23/310 (7.4) | 25/286 (8.7) | 1.2 (0.7–2.2) | | 32/325 (9.9) | 1.4 (0.8–2.2) | |
| Respiratory syncytial virus infection | 32/463 (6.9) | 14/331 (4.2) | 0.6 (0.3–1.1) | | 22/418 (5.3) | 0.7 (0.4–1.3) | |
| Adenovirus infection | 99/463 (21.4) | 24/331 (7.3) | 0.3 (0.2–0.5) | | 69/418 (16.5) | 0.7 (0.5–1.0)§ | |
| Parainfluenzavirus 1, 2, or 3 infection | 14/463 (3.0) | 13/338 (3.9) | 1.3 (0.6–2.8) | | 17/418 (4.1) | 1.4 (0.7–2.8) | |
| Human metapneumovirus infection | 6/463 (1.3) | 8/331 (2.4) | 1.9 (0.6–5.5) | | 4/418 (1.0) | 0.7 (0.2–2.6) | |
| Rhinovirus infection | 61/462 (13.2) | 30/331 (9.1) | 0.7 (0.4–1.0)§ | | 57/418 (13.6) | 1.0 (0.7–1.5) | |
| Enterovirus infection | 16/463 (3.5) | 5/331 (1.5) | 0.4 (0.2–1.2) | | 12/418 (2.9) | 0.8 (0.4–1.8) | |
| Clinical presentation and course | | | | | | | |
| Temperature ≥38°C | 181/364 (49.7) | 141/287 (49.1) | 1.0 (0.7–1.3) | | 138/407 (33.9) | 0.5 (0.4–0.7) | |
| Cough ¶ | 255/264 (96.6) | 162/167 (97.0) | 1.1 (0.4–3.5) | | 163/170 (95.9) | 0.8 (0.3–2.2) | |
| Tachypnea¶ | 99/250 (39.6) | 73/161 (45.3) | 1.3 (0.8–1.9) | | 62/159 (39.0) | 1.0 (0.6–1.5) | |
| Difficulty breathing¶ | 188/264 (71.2) | 125/167 (74.9) | 1.2 (0.8–1.9) | | 111/170 (65.3) | 0.8 (0.5–1.1) | |
| Chest wall indrawing¶ | 96/264 (36.4) | 77/167 (46.1) | 1.5 (1.0–2.2)§ | | 56/170 (32.9) | 0.9 (0.6–1.3) | |
| Stridor¶ | 30/264 (11.4) | 20/167 (12.0) | 1.1 (0.6–1.9) | | 36/170 (21.2) | 2.1 (1.2–3.6) | |
| Tachycardia¶ | 132/264 (50.0) | 95/167 (56.9) | 1.3 (0.9–1.9) | | 88/170 (51.8) | 1.1 (0.7–1.6) | |
| Diarrhea¶ | 50/264 (18.9) | 28/167 (16.8) | 0.9 (0.5–1.4) | | 32/170 (18.8) | 1.0 (0.6–1.6) | |
| Unable to eat¶ | 66/264 (25.0) | 43/167 (25.8) | 1.0 (0.7–1.6) | | 35/170 (20.6) | 0.8 (0.5–1.2) | |
| Vomiting¶ | 70/264 (26.5) | 52/167 (31.1) | 1.3 (0.8–1.9) | | 53/170 (31.2) | 1.3 (0.8–1.9) | |

| | Influenza type and subtype | | | | | | |
|---|---|---|---|---|---|---|---|
| | A(H3N2) (reference) | A(H1N1)pdm09 | | | B | | |
| Characteristic | No. pos/no. tested (% pos) | No. pos/no. tested (% pos) | Crude RRR (95% CI) | Adjusted RRR (95% CI) | No. pos/no. tested (% pos) | Crude RRR (95% CI) | Adjusted RRR (95% CI) |
| Lethargy¶ | 53/264 (20.1) | 32/167 (19.2) | 0.9 (0.6–1.5) | | 30/170 (17.7) | 0.9 (0.5–1.4) | |
| Symptoms ≥3 d before admission | 206/452 (45.6) | 153/335 (45.7) | 1.0 (0.8–1.3) | | 239/415 (57.6) | 1.6 (1.2–2.1) | |
| Admission to intensive care unit | 4/457 (0.9) | 3/336 (0.9) | 1.0 (0.2–4.6) | | 4/411 (1.0) | 1.1 (0.3–4.5) | |
| Mechanical ventilation needed | 3/457 (0.7) | 1/336 (0.3) | 0.5 (0.0–4.4) | | 4/411 (1.0) | 1.5 (0.3–6.7) | |
| Supplemental oxygen needed | 138/457 (30.2) | 117/336 (34.8) | 1.2 (0.9–1.7) | | 144/411 (35.0) | 1.2 (0.9–1.7) | |
| Antimicrobial drugs prescribed at admission | 402/421 (95.7) | 321/335 (95.8) | 1.0 (0.5–2.1) | | 384/395 (97.2) | 1.6 (0.7–3.4) | |
| Duration of hospitalization ≥2 d | 319/451 (70.7) | 255/332 (76.8) | 1.4 (1.0–1.9)§ | | 323/407 (79.4) | 1.6 (1.2–2.2) | |
| Case-fatality ratio, no. who died | 13/459 (2.8) | 5/334 (1.5) | 0.5 (0.2–1.5) | | 16/412 (3.9) | 1.4 (0.7–2.9) | |

*Pos, positive; RRR, relative risk ratio.
†p<0.05.
‡Asthma, other chronic lung disease, chronic heart disease (valvular heart disease, coronary artery disease, or heart failure excluding hypertension), liver disease (cirrhosis or liver failure), renal disease (nephrotic syndrome, chronic renal failure), diabetes mellitus, immunocompromising conditions excluding HIV infection (organ transplant, immunosuppressive therapy, immunoglobulin deficiency, malignancy), neurologic disease (cerebrovascular accident, spinal cord injury, seizures, neuromuscular conditions), or pregnancy. Concurrent conditions were considered absent for patients for whom the medical records stated that the patient had no underlying medical condition or when there was no direct reference to that condition.
§p>0.05.
¶Patients <5 y of age.
